# Machine Learning and Radiomics Applications in Esophageal Cancers Using Non-Invasive Imaging Methods—A Critical Review of Literature

**DOI:** 10.3390/cancers13102469

**Published:** 2021-05-19

**Authors:** Chen-Yi Xie, Chun-Lap Pang, Benjamin Chan, Emily Yuen-Yuen Wong, Qi Dou, Varut Vardhanabhuti

**Affiliations:** 1 Department of Diagnostic Radiology, Li Ka Shing Faculty of Medicine, The University of Hong Kong, Hong Kong, China; chenyix@hku.hk; 2 Department of Radiology, The Christies’ Hospital, Manchester M20 4BX, UK; c.pang@doctors.org.uk; 3 Division of Dentistry, School of Medical Sciences, University of Manchester, Manchester M15 6FH, UK; 4 Li Ka Shing Faculty of Medicine, The University of Hong Kong, Hong Kong, China; chanbenj@connect.hku.hk (B.C.); emily372@connect.hku.hk (E.Y.-Y.W.); 5 Department of Computer Science and Engineering, The Chinese University of Hong Kong, Hong Kong, China; qidou@cuhk.edu.hk

**Keywords:** machine learning, esophageal neoplasms, radiology

## Abstract

**Simple Summary:**

Non-invasive imaging modalities are commonly used in clinical practice. Recently, the application of machine learning (ML) techniques has provided a new scope for more detailed imaging analysis in esophageal cancer (EC) patients. Our review aims to explore the recent advances and future perspective of the ML technique in the disease management of EC patients. ML-based investigations can be used for diagnosis, treatment response evaluation, prognostication, and investigation of biological heterogeneity. The key results from the literature have demonstrated the potential of ML techniques, such as radiomic techniques and deep learning networks, to improve the decision-making process for EC patients in clinical practice. Recommendations have been made to improve study design and future applicability.

**Abstract:**

Esophageal cancer (EC) is of public health significance as one of the leading causes of cancer death worldwide. Accurate staging, treatment planning and prognostication in EC patients are of vital importance. Recent advances in machine learning (ML) techniques demonstrate their potential to provide novel quantitative imaging markers in medical imaging. Radiomics approaches that could quantify medical images into high-dimensional data have been shown to improve the imaging-based classification system in characterizing the heterogeneity of primary tumors and lymph nodes in EC patients. In this review, we aim to provide a comprehensive summary of the evidence of the most recent developments in ML application in imaging pertinent to EC patient care. According to the published results, ML models evaluating treatment response and lymph node metastasis achieve reliable predictions, ranging from acceptable to outstanding in their validation groups. Patients stratified by ML models in different risk groups have a significant or borderline significant difference in survival outcomes. Prospective large multi-center studies are suggested to improve the generalizability of ML techniques with standardized imaging protocols and harmonization between different centers.

## 1. Introduction

Esophageal cancer (EC) is a malignancy affecting more than 500,000 people worldwide, ranking as the sixth leading cause of cancer death [1,2]. ECs are classified based on cell types on histopathology, most commonly identified as either adenocarcinoma or squamous cell carcinoma (SCC). Late presentation is a frequent occurrence which means a higher proportion of EC patients have a worse prognosis [2]. The standard treatments for EC patients include surgery, radiotherapy, and chemotherapy. It is reported that neoadjuvant chemoradiotherapy (nCRT) plus surgery can improve patient prognosis for late-stage EC [3,4]. Pathologic complete response (pCR) is related to the improvement of patients’ survival. However, due to tumor heterogeneity, a proportion of patients receiving nCRT may still have disease recurrence after treatment [5]. Unfortunately, some patients may fail to achieve sufficient tumor reduction prior to surgery as well as suffer from chemoradiation-related toxicity. For locally advanced disease, chemoradiotherapy is considered an option to improve survival and reduce recurrence [6]. Despite recent advances in therapeutics, the 5-year overall survival rate remains poor, ranging from 15% to 25% (3).

Accurate staging, treatment planning, and prognostication in EC patients is vital. Most recently, investigators have looked at novel applications such as radiomics using non-invasive imaging methods to improve the patient pathway. Previously hidden information can be found among different imaging modalities which may reflect the pathogenesis of EC. Computed tomography (CT), positron emission tomography (PET), mostly combined with CT (PET-CT), magnetic resonance imaging (MRI), and endoscopic ultrasonography (EUS) are commonly used for staging and follow-up [7]. The two most commonly used modalities for EC patients are CT and PET. However, their abilities to find small-sized lesions are limited, which affects the sensitivity and specificity of disease detection [8].

Artificial intelligence (AI) is a general term that refers to a wide range of algorithms capable of identifying features amongst a large amount of data and performing inferences. Machine learning (ML) is a subset of AI and refers to algorithms able to learn and predict without explicit instructions. The development of ML has provided a new scope in imaging analysis. Radiomics can convert images into high-dimensional features by calculations based on shape and textural features as well as higher-order spatial features, incorporating relationships at a pixel level [9]. It has been reported that using a handcrafted radiomics approach improved the imaging-based classification system in characterizing the heterogeneity of primary tumors and lymph nodes in EC patients. More recently, deep neural networks, a branch of ML, have been regarded as promising alternatives for extracting features and conducting medical image analysis in an end-to-end scheme [10,11,12]. Remarkable success with various analysis tasks have been witnessed, including diagnosis of skin cancer [13], prediction of cardiovascular risk factors [14], classification of lung cancer histopathology images [15], detection of lung abnormalities in COVID-19 CT images [16], and automated segmentation for robust quantitative measurements [17]. Unlike the conventional handcrafted radiomics approaches, deep networks are composed of multiple nonlinear transformation layers to perform automatic feature extraction in a data-driven way, thus is powerful in capturing more high-level and generalizable features for intelligent analysis of complex medical images. Nevertheless, there is ongoing debate, particularly about the interpretability and clinical usefulness of ML studies. We set out to provide a summary of the evidence of the most recent developments in ML application in imaging pertinent to EC diagnosis and follow-up.

## 2. Machine Learning and Radiomics Workflow for Oncology Imaging

### 2.1. Radiomics

Advancements in pattern recognition, medical image analysis, and artificial intelligence have laid the foundation for the rapid maturation of radiomics. Radiomics is the process of quantifying medical images into high-dimensional data with respect to prediction targets and subsequently mining the data for more informed clinical decision-making [18]. Contrary to conventional application of medical images, mainly for qualitative diagnosis and visual representation, radiomics exploits image analysis to identify features, often from standard-of-care images, for improving prediction accuracy in diagnosis, prognosis, and determination of optimal treatment regimes. Inclusion of the suffix -omics in radiomics indicates its ability in producing high-dimensional data from limited samples, as -omics fields are characterized by their data-mining potential [19]. The idea that studying information embedded in medical images could aid in improving prediction and reflect more accurate clinical endpoints is the driving force of radiomics. More specifically for cancer imaging, extracting image features based on tumor texture, phenotype, and habitat, radiomics’ potential in quantifying intratumoral and intertumoral heterogeneity brings hope for more personalized and effective treatments for cancer patients, particularly where contemporary treatments may fail due to intratumor heterogeneity [20]. Varying methods of feature extraction exist between different radiomics pipelines [21]. The image biomarker standardization initiative (IBSI) is an independent international collaboration, which aims to improve the reproducibility and standardization of radiomics studies [22]. The IBSI reference manual has made recommendations for a set of standardized radiomics feature calculation and processing guidelines to practice radiomic studies. It is suggested to check radiomics software’s compliance with the IBSI standard before application for better clinical translation.

### 2.2. Radiomics Workflow

The workflow of a radiomic study can be categorized into six stages: problem identification, data curation, feature extraction, feature reduction, modelling, and model validation [23,24].

#### 2.2.1. Problem Identification

The first step is to identify the clinical problem and what researchers are aiming to predict. A prediction endpoint (PE) usually refers to a clinical endpoint relevant to the patient and attending clinician. PEs could be categorical (for example treatment response patterns) or scaler (for example disease-free survival in months).

#### 2.2.2. Data Curation

Information about imaging modality that would be applicable to the clinical problem is needed. Image acquisition follows and involves obtaining the medical images from all patients in full accordance with the protocol to ensure consistency. Radiomics features are typically extracted from the regions of interests (ROIs). Delineation of ROI is conducted manually, semi-automatically or fully automatically. Manual segmentation requires experienced radiologists to contour the full tumor regions, which is still regarded as the gold standard in many applications [25,26,27]. Semi-automatic segmentation is usually realized by thresholding and region-growing techniques, which is more efficient as it requires less manual intervention [28,29]. The manual or semi-automatic segmentation could be conducted using open-source or commercial software, such as ITK-SNAP [30], LIFEx [31], MITK [32], ImageJ [33], and 3D Slicer [34]. Bias caused by inter-algorithm and inter-reader variability [35] could be limited by multiple segmentation, such as manual segmentation by multiple clinicians or using a diverse combination of segmentation algorithms. A recent systematic review by Traverso et al. [36] pointed out that radiomics textural and shape features could be sensitive to manual segmentation differences. Semiautomated segmentation methods are found to provide a better alternative to manual slice-by-slice delineations in terms of feature reproducibility [37,38]. Automatic segmentation methods are suggested to be broadly applied in EC studies to optimize the clinical applicability. Auto-segmentation using deep learning is now a rapidly developing technique and has been proved to be potentially a reliable and reproducible tool for tumor delineation [39,40]. U-Net is a commonly used network architecture for auto-segmentation in medical images with exceptional performance demonstrated on many applications, which is a convolutional neural network consisting of a contracting path and a symmetric expanding path [41]. More complex deep neural network architectures with residual connections, skip connections, dense connections, or attention mechanisms have also been investigated for boosting automated segmentation accuracy. In addition to medical imaging data, researchers will also need to collect related clinical data (for example survival outcomes, demographics, clinical categorization).

#### 2.2.3. Feature Extraction

Feature extraction with high throughput is the essence of radiomics and involves quantitative descriptions of characteristics of ROI. There are several features that can be extracted, including shape-based features, first order, and higher-order features. Currently, there are several open-source packages and software available that can be utilized to create up to thousands of features. Readers are advised to refer to the following excellent articles for further insight [22,31,42]. It is important to note that features can be computed based on pixel spacing, quantization method, and the number of gray levels but care must be taken to ensure a consistent approach is taken when generating these features. Moreover, radiomics features could be both 2-dimensional (2D), depicting the lesion in a representative layer (usually the layer with the maximal diameter of largest tumor cross-section) or 3-dimensional (3D), contouring the whole lesion volume ROIs. Controversy still exists over the discriminative ability of 2D and 3D radiomics features. The 3D ROIs are considered to contain more comprehensive information and have shown good classification ability [43]. However, Shen et al. has demonstrated that 2D radiomics features showed better prognostic performance than 3D features of non-small cell lung cancer, probably due to inconsistent spatial resolution in CT images [44]. Yang et al. has proved that a combination of 2D and 3D features could optimize the prediction performance [45].

Radiomics features are sensitive to CT acquisition and reconstruction parameters. It is impractical to standardize platforms and parameters from all institutions in clinical settings. To reduce the batch effect, the ComBat harmonization has been recently shown to correct the difference in extracted radiomics feature values resulting from different image acquisition protocols [46,47]. This method has been used to properly correct for the scanner effect while preserving the specific characteristics of each texture pattern.

#### 2.2.4. Feature Reduction

Increasing the number of features does not necessarily mean better performance. In fact, it has been shown that a high number of features often leads to high redundancy and poor repeatability necessitating feature reduction [48]. The purpose of feature reduction is to optimize the feature space (either to selected few from the original list, engineered, or by reducing the dimension) before inputting into the model.

Feature selection is a common method used to remove irrelevant and redundant features. Traditionally, they are divided into filter, embedded, and wrapper methods. Filter methods have been most favored, and these are independent of the machine learning classifier. A subset class of the filter method known as ranker filters are techniques that have been most commonly employed in the early machine learning application in radiomics. In practical terms, the process usually begins with the removal of correlated features, usually based on a correlation matrix. For example, a Pearson’s correlation matrix can be computed on the features set, and the highly correlated features (set for example as *p* > 0.95) can then be excluded. Association scores can further be created based on univariate or multivariate analyses and these can be ranked according to the association scores. A common method is to then choose the top-ranked features (either in number or proportion). Additionally, features can also be subsequently ranked based on their predictive power, for example using Maximum Relevance Minimum Redundancy (mRMR) and recursive feature elimination (RFE) [49,50]. Care must be taken when using other feature selection methods such as wrapper selection bias could be introduced, although this could be mitigated by ML approaches such as Borda method or the bootstrap method [51,52]. Investigators have also used regularization techniques. Regularization techniques, such as least absolute shrinkage and selection operator (LASSO) and ridge regression in prediction models, aim to reduce (or shrink or smooth) the number of input features by removing redundant or highly correlated features [53]. Finally, investigators have also used unsupervised dimension reduction methods that could reduce feature number without the introduction of PE information, for example principal component (PCA) or linear discriminant analysis (LDA). These have been shown to reduce overfitting and improve prediction performance in ML models [54,55]. Reducing the number of features helps with interpretation, making a more plausible linkage between feature and outcome easier to identify.

#### 2.2.5. Modelling

Model development aims to classify patients into different risk groups in relation to PE. ML methods explore the correlation between selected features and the targeted PE in the training set [25]. *Logistic regression* is a classic binary classification tool that fits a logistic function to classify data into one of two dependent variables and can be extended to classify multiple groups of events [56]. *Support vector machine* is one of the most commonly used ML models for radiomics studies [49,57,58]. This technique identifies a hyperplane or set of hyperplanes in the high-dimensional feature space for the classification of new data [59]. *K-nearest neighbor* classifies an unknown datum by comparing it with labelled data [60]. *Decision trees* are a series of classification branches that help visualize the process of making a prediction about the PE [61]. *Random forests* are a combination of multiple *decision trees* developed from the same training set to correct for potential overfitting of using a single decision tree [62]. Previous studies comparing the predictive performances across different classifiers have shown the optimal performance achieved by *Random forests* [63,64].

Overfitting may result from complex model architectures which have the issue of over-parameterization. The model may perform well in training data, but poorly in validation data, even split from the same cohort source. This can be reduced by acquiring sufficient data for training or making the training data more diverse so that it can be representative of the population. If the rate of the expected outcome is low making the dataset imbalanced, there are resampling techniques that may help to boost the minority class and improve predictive performance [63,65]. While it is ideal to keep the number of features as low as possible, the number of features is not a major issue if the model is validated externally with reasonable performance [18]. The selection of the optimal model for one specific dataset requires an understanding of the classifier mechanism and characterization of the dataset or exploratory data experiments. For better clinical application, a nomogram incorporating the radiomics signature and clinical risk factors could be built. This graphical calculation instrument is a user-friendly tool for patient-clinician communication [66]. Akaike information criterion (AIC), which measures the goodness of fit of the model, is commonly used for selection of important clinical variables for the final nomogram model [67].

#### 2.2.6. Model Development

In machine learning model development, it is preferred for the input dataset to be divided into the training set and the test set. A “training set” is only used to train the model. A “test set” could be further divided into internal or external, and is only used to evaluate the model. If the test set is from an independent center, this is often termed “external validation” set in clinical studies. This means that there is no overlap with the training data, and it is also from a different center. Nonetheless, it may be difficult to recruit significant amounts of independent data in preliminary or pilot radiomics studies. If this is not possible, then internal validation method of the test set is used. Note that the term “internal validation has different meanings in clinical and machine learning literatures. For the purpose of this article “internal validation” refers to validation with the dataset from one single center.

In some cases, data can be used for model hyper parameters optimization requiring “cross-validation”, in which part of the training dataset is used for training and part for validation. There are several methods to do this. Firstly, in k-fold cross-validation [13], the dataset is divided into k (usually k = 10) groups (called folds). (k-1) groups will be used as the training set whilst the remaining group is the testing set. A small proportion of data from the training set is used to validate the model during training time, in order to select hyper-parameters of ML models. This process is repeated k times in the test set once and training set k-1 times, with each group being in the training set k-1 times and testing set once. This ensures that all data is used to test the model for performance evaluation. Secondly, in leave-one-out cross-validation [68], which is similar to k-fold cross-validation, the data is divided such that each patient is treated as one fold. In each cycle, one patient will be the testing set whilst all others will be the training set; this process repeats until all patients have been used in the testing set once. This scheme is used only when the dataset is small, in order to keep more data for model training. Thirdly, hold-out validation [69], which is the simplest form of internal validation in one center, involves randomly separating the data into a training set (again including part of data for model hyper-parameter validation) and a testing set [70]. Besides these, a more ideal way is to validate the model in a prospective dataset.

Several performance metrics have been used in the evaluation of ML performance, and a few commonly used methods will be discussed here. The most commonly used method typically involves an analysis of the area under curve (AUC) of the receiver operating characteristic (ROC) curve. Comparison between AI algorithms can be done by evaluating the AUC and individual sensitivity, specificity values of each ROC. The optimal AI algorithm depends on the context in which the model will be implemented. For comparison between humans and AI performance, a summary ROC curve rather than the convention of using one point on an ROC graph can more accurately quantify human performance and allow for robust comparison between ML models and humans [71]. Other commonly used metrics such as Matthew’s correlation coefficient (MCC) have also been used which incorporates true and false positives, negatives, and equals [72]. A coefficient of +1 represents a perfect prediction, 0 no better than chance, and −1 total disagreement between prediction and the actual outcome. Precision-Recall (PR) curve has also been used, which plots precision against recall to show the trade-off between them for the different feature or parameter settings. For classification tasks involving datasets with very few positive cases, the area under the PR curve (AUPRC) is a better measure of accuracy than AUROC [73].

Validation reflects the discrimination and calibration capacity of the model, which respectively quantifies model sensitivity and specificity, and the agreement between predicted and observed outcomes. Relying purely on the performance metrics outlined above may have limitations. For example, risk prediction models may be highly discriminatory but poorly calibrated [74] or have sensitivity/specificity cut-points that fail to maximize clinical utility [75].

### 2.3. Deep Learning

Different from the conventional low-level radiomics features with hand-crafted descriptors, deep learning [76] has recently emerged to automatically learn high-level features in a data-driven way, which is capable of capturing the complicated characteristics of medical images. Among different classes of deep learning algorithms, convolutional neural network is one of the most widely-used solutions in analyzing image data to extract hierarchical feature representations. A convolutional neural network (CNN) is composed of multiple convolutional layers with a set of filters (also called kernels), which is the core component, combining with pooling operations and non-linear activation functions. The weight-sharing of convolution kernels over the entire input image significantly reduces the number of trainable parameters. CNNs with 2D kernels (namely 2D CNNs) are generally applicable to different types of imaging modalities, but for volumetric images, 3D CNNs have been demonstrated to be more effective in capturing important contextual information along the third dimension [77,78].

For recent deep learning models, the classifier learning can be performed in a unified network with the feature extraction and trained in an end-to-end process. As previously mentioned, CNNs are the most popular deep networks in medical image analysis and have shown exceptional performance. Alexnet [79] is one of the commonly used CNN architectures in cancer imaging [80], which is an early proposed shallow network with 11 layers. Inception network [81] is another popular architecture [13], which has a more complex design with deeper architecture and multiple filters of different sizes operating on the same level. ResNet [82] is another recent CNN architecture with skip connections to benefit gradient backpropagation [83]. Besides using standard network architectures, some studies have designed specific CNNs for the targeted problem. For example, Bizzego et al. [72] use two identical and parallel CNN streams for CT and PET data respectively, that each stream consisting of five convolutional layers combining with normalization, dropout, and pooling layers, and the outputs of the two streams are merged at the fully connected layer. Effective training of a CNN usually requires large amounts of data to sufficiently optimize the large number of parameters in a network and to reduce the overfitting problem. If the size of available dataset is large enough, training a deep network from scratch can better optimize the model for the specific problem and dataset. However, obtaining annotations from medical experts is costly and typically limits the amount of available data for network training. When data is limited, data augmentation techniques, such as generative or associative modelling can generate additional, artificial training data based on real training sets [84,85].

Transfer learning is commonly adopted to mitigate the problem of inadequate training data. With transfer learning, a deep network is firstly trained with available large-scale natural images or medical images of different problems, the pre-trained network is then fine-tuned with the desired data and task. The effectiveness of transfer learning in reducing the overfitting problem caused by a small number of training samples and improving the model’s performance has been well studied in previous works on mammographic tumor classification [86] and thoraco-abdominal lymph node detection [87]. Deep features can be extracted from medical images by training CNNs with pre-defined prediction tasks such as disease classification, tumor stage diagnosis, or survival prediction. For deep learning-based radiomics with end-to-end training, the extracted features can be directly used by the classifier layer of the rest of the deep networks for the targeted analysis tasks. There are also studies separating the learned features from the deep networks to be combined with the hand-crafted radiomics features for subsequent modelling [72]. The major advantage of deep learning-based feature extraction is that no specific domain knowledge is required for feature engineering, and the representative and high-level features can be learned in a completely automatic manner. Recent works [88,89] have shown that the automatically learned deep features of neural networks can outperform the hand-crafted ones in some applications. One key challenge of applying deep networks in clinical decision making is that deep networks are black box models with multilayer nonlinear operations, thus the reasoning behind the results from deep networks are very difficult to interpret clinically. Explainable AI is an emerging field of active research in trying to address this challenge [90,91].

Recent studies [72,80] have shown that the conventional handcrafted radiomics and the deep learning-based radiomics can be fused to improve analysis accuracy. The fusion operation can be performed at the decision level or at the feature level. For decision-level fusion, the hand-crafted radiomics and deep radiomics are first independently trained to produce prediction respectively, then the outputs are then combined together with a certain voting strategy to achieve the final decision. For feature-level fusion, the hand-crafted features and deep features are extracted separately, and then the two types of features are combined to go through a classifier for the final prediction. On the other hand, the combination of handcrafted radiomics and deep learning features did not enhance the prediction performance in Yun’s previous study [92]. This is an area of active research, and further investigations are needed to properly address the value of radiomics in combination with deep learning methods [93]. The use of deep learning methods, however, is advantageous in potentially being more automated than radiomics features extraction, as the latter requires segmentation, which depending on the disease entity may not be automated.

### 2.4. Automation of Machine Learning Pipeline in Clinical Workflows

For the identification of optimal pattern for a specific dataset, the discovered machine learning pipeline could be quite variable, as the selection of the ML approach and setting of parameters are dependent on individual researchers. In recent years, there has been a trend for automation of machine learning pipeline in clinical workflows. Cai et al. [94] provided an online calculator based on the radiomics model for the prediction of treatment response of bevacizumab in brain necrosis after radiotherapy. Such a user-friendly tool could help the facilitation of personalized and precise treatment of patients. Su et al. [95] applied the Tree-based Pipeline Optimization Tool for the construction of the optimal radiomics model. Without human annotation, the automatically optimized machine learning pipeline showed good prediction accuracy for H3 K27M mutation status in patients with midline gliomas. More recently, automation of machine learning pipeline has found promising applications for fast diagnosis and risk stratification for patients with infectious diseases [96,97,98]. Wang et al. [96] developed a novel fully automatic deep learning system using CT imaging for fast screening of COVID-19 to identify potential high-risk patients, which could be fast and more repeatable as it required no time-consuming human involvement. Automation of machine learning pipeline in clinical workflows could significantly improve the optimization process of medical resource.

## 3. A review of Literature Using Machine Learning and Radiomics Applications in EC

### 3.1. Eligible Studies

In this paper, a comprehensive review of studies based on ML methods for the diagnosis of any aspect in EC patients using non-invasive medical imaging was performed. According to the scope of the review, we consider ML-based investigations aimed at relevant objectives in clinical practice: treatment response evaluation, prognostication prediction, diagnosis, and biological characterization. Non-invasive imaging modalities considered are CT, PET, PET-CT, and MRI. Studies using invasive endoscopy or endoscopic ultrasound were not included. We searched for articles in 3 database resources including PubMed, EMBASE and Cochrane Library. All the English publications from 1 January 2000 until 16 October 2020 were searched. The reference articles of the selected papers were also checked. The complete search strategy is shown in Supplementary Material. We included any study design, with a minimum of 10 patients, except for letters to the author, comments, and case reports. Studies with only correlation analyses between individual imaging features and outcomes, without utilizing ML approaches were excluded.

### 3.2. Data Analysis

The measurements obtained in the validation groups were used as the main results. For papers with multiple clinical outcomes, the primary aim was chosen. The AUC was preferred to summarize the predictive value of proposed models. If unavailable, other accuracy metrics were recorded (for example sensitivity, specificity, C-index, etc.). We rated AUCs by the following range: 0.60–0.70 as poor, 0.70–0.80 as acceptable, 0.80–0.90 as excellent, and 0.90–1.00 as outstanding [99]. For prognostic groups, cox-proportional hazards ratios were used as outcome measures for risk stratification. For articles with multiple test models for the same cohort of subjects, the final model with the best performance was recorded. Confidence intervals of these accuracy metrics were retrieved if available.

**Figure 1:**
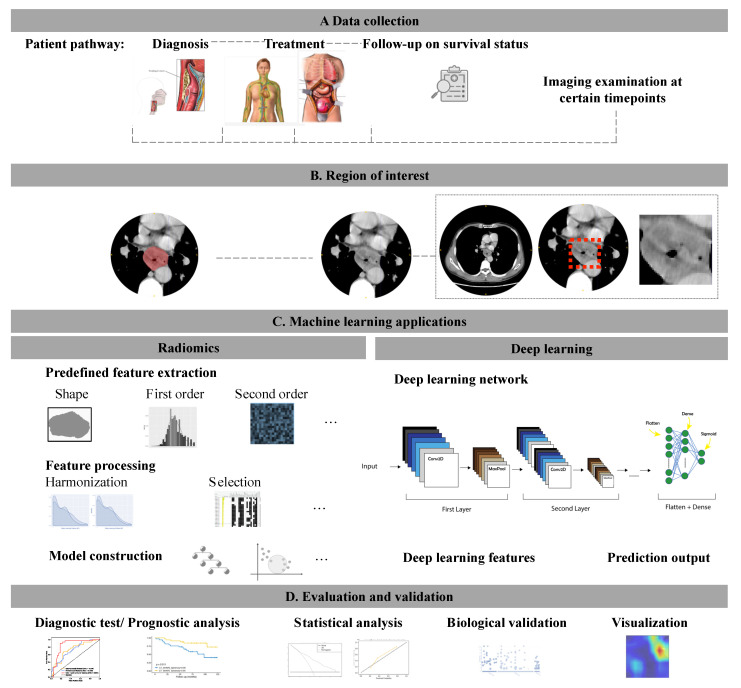
An analysis workflow summarization of studies for imaging machine learning applications in esophageal cancers. (**A**) Patient pathway in clinical practice (**B**) Radiological features extracted from the handcrafted radiomics and deep learning method using different regions of interest. (**C**) Machine learning models constructed with the selected features (**D**) Model evaluation and evaluation.

The results from the included studies are summarized in Table 1. The studies for imaging machine learning applications in EC commonly followed certain steps, which are shown in Figure 1.

### 3.3. Main Findings

#### 3.3.1. ML and Treatment Response Evaluation in ECs

Most published studies (*n* = 12) focused on the prediction of treatment response for patients receiving chemoradiotherapy or nCRT [83,100,101,102,103,104,105,106,107,108,109,110]. The ML algorithms achieved an AUC of 0.78–1.00. There were six studies using PET, four studies using CT, one study using MRI, and one study using PET-CT. Zhang et al. [110], which constructed a support vector machine model based on PET radiomics features combined with conventional imaging and clinical variables. They achieved the best prediction result (AUC = 1.00) with 20 patients although with no validation on a test set. Ypsilantis et al. [109] provided initial evidence for the potential predictive power of CNNs with an averaged sensitivity and specificity of 80.7% and 81.6%, respectively. Desbordes et al. [108] applied a random forest classifier based on conventional and textural features for treatment response prediction by using baseline PET images. Both individual features and the constructed model with combined features were of predictive value, with an AUC of 0.810 and 0.836. Beukinga et al. published two studies evaluating radiomics for the prediction of response to nCRT. The first study [107] selected only pre-treatment clinical and radiomics features from PET/CT scans. For the latter study [104], significant features were derived from PET images of two phases (both baseline and restaging). The discrimination accuracy was improved from acceptable (AUC = 0.78) to excellent (AUC = 0.81). Van Rossum et al. [106] also showed the added value of posttreatment radiomics features for disease evaluation. Hou et al. studied two modalities for the evaluation of CRT response. Radiomics features extracted from both the CT [105] and MRI [103] showed predictive capabilities with AUC of 0.972 for CT and AUC of 0.929 for MRI, using imaging interpretation as a reference standard. Yang et al. [102] developed three predictive models for treatment response after nCRT and noted that overfitting was a problem for small sample size studies. Hu et al. [101] found that a combination of peritumoral radiomics features appeared to improve the predictive performance of intratumoral radiomics for pre-treatment prediction of pCR (AUC = 0.85, 95% CI, 0.75–0.95) for nCRT using CT radiomics features, with a cohort of 231 patients and external validation of the results. The same cohort of patients was used for the exploration of the transfer learning approach and the results showed that ResNet50-based deep learning features had the predictive ability for treatment response in esophageal SCC [83]. Cao et al. [100] used the limma method, which is commonly used for genetic analysis, for the identification of significant radiomics features. The selected features were then fitted into the least absolute shrinkage and selection operator (LASSO) logistic regression model and achieved an AUC of 0.84 in the test set to predict response for concurrent CRT. The majority of studies [83,101,102,104,106,107,109,110] evaluated pCR for nCRT before surgery and used histology results as the reference standard. Other studies [100,103,105,108] collected patients receiving chemoradiotherapy and adopted PET/CT/MRI imaging and follow-up information for pCR evaluation because surgical resection was not performed and, therefore, pathological reference was not available.

#### 3.3.2. ML and Prognosis Prediction in ECs

ML methods have shown the potential to act as prognostic tools for risk stratification in ECs. There are seven studies in the prognostic group [111,112,113,114,115,116,117], including three PET, three CT, and one PET-CT studies. Patients in different risk groups had a significant or borderline significant difference in survival outcomes. The primary outcomes were overall survival (OS), recurrence-free survival (RFS), and disease-free survival (DFS) predictions. Xiong et al. [117] extracted PET radiomics features from different treatment time points and concluded that mid-treatment features could be more informative for predicting local control. Foley et al. [116] demonstrated that a clinical prognostic model incorporating PET radiomics features could bring additional benefit for patients’ risk stratification. Larue et al. [115] validated their radiomics-based model for EC patients receiving nCRT in an independent external cohort, which split risk groups for survival rates with borderline significance. Xie et al. [114] found that sub-regional radiomics features were of prognostic prediction value. The authors further correlated the imaging traits with the clinical variables and gene data to verify the biological significance. Yang et al. [113] proved that deep learning-based prediction could be an independent survival prognosticator for EC patients. Chen et al. [112] constructed a scoring system based on both clinical and PET radiomics features and enabled better stratification of patients into different long-term prognosis. Qiu et al. [111] developed three prognostic models, the nomogram based on both radiomics and clinical features achieved optimal performance with a C-index of 0.72 in the validation set.

#### 3.3.3. ML and Lymph Node Metastasis Status in ECs

There were four studies [88,118,119,120] focusing on the lymph node metastasis status of EC, with three studies using CT and one study using MRI. Shen et al. [120] built a nomogram incorporating radiomics features, CT-reported suspicious lymph node number and tumor location, which showed good discrimination of lymph node status with a C-index of 0.75 in the validation set. Tan et al. [119] demonstrated that radiomics nomogram provided a good estimation of lymph node metastasis (AUC = 0.77) and outperformed size criteria. Most studies delineated whole tumor volume in all slices as the ROIs for feature selection. Qu et al. [118] provided evidence that MRI-based radiomics features were of predictive value for lymph node involvement. Wu et al. [88] showed that two-dimensional (2D) ROIs based on the largest cross-sectional area of the tumor lesion were also of prediction value (AUC = 0.84).

#### 3.3.4. ML and Other Clinically Significant Outcomes in ECs

There were two studies focusing on the diagnosis of EC and one study focusing on gene expression [121,122,123]. CT imaging was used. Li et al. [121] found differences in the same parts of the normal esophageal wall and EC lesions using a multivariate regression classifier based on radiomics features. Ou et al. [122] investigated the value of radiomic models related to the resectability of ECs. Hoshino et al. [123] found that the gene expression level of miR-1246 could be inferred by imaging radiomics features, which was predictive of the prognosis of EC patients.

#### 3.3.5. Study Characteristics

In general, all studies were retrospective design and most studies adopted radiomics approaches. Four studies [88,109,113,115] applied deep learning networks in the model construction. Incorporation of clinical features into data-based ML models could improve the prediction accuracy in 11 studies [88,104,106,110,111,112,113,116,119,120]. The majority of studies [83,88,100,101,102,103,105,111,112,113,114,119,121,122,123] evaluated SCC patients and some [104,106,107,108,109,110,115,116] mainly focused on adenocarcinoma. Many studies (*n* = 11) did not provide sufficient sample size with less than 100 patients in the study cohort [102,103,104,105,107,108,110,112,117,121,123]. Four studies [104,107,121,123] lacked independent validation by using the same datasets as both training and test sets.

Feature harmonization was conducted in only two studies to remove inter-site technical variability [83,101]. Nine studies [88,103,104,106,111,116,119,120,122] applied additional statistical analysis on the prediction outcomes to evaluate model fitting, calibration, and clinical usefulness. Three studies [83,101,114] explored the correlation between imaging features extracted by ML and the genomic profiles for biological interpretation. Two studies [83,109] used visualization techniques to highlight important regions in the medical images. The visualized pictures generated from specific layers of the deep learning model highlighted the regions of interest for patient characterization. Visualizing the hot zone in the feature map could help to evaluate the interpretability of the models.

## 4. Summary and Perspectives

This study summarizes the main results and basic characteristics of ML techniques relevant to the clinical practice in EC patients. Surgery is one of the most frequently used treatments for resectable EC, but the long-term survival remains unsatisfactory even after curative operation [124,125]. The optimal treatment for EC is still unclear. Neoadjuvant or adjuvant therapies administrated as chemotherapy, radiotherapy, or simultaneous chemoradiotherapy have been adopted in clinical practice. The adjuvant chemotherapy or radiation therapy has not been shown to have additional survival benefit to patients compared to surgery alone [126]. More recently, as shown by recent landmark trials, nCRT plus surgery could be the most effective strategy for the improvement of resectability and maximization of long-term survival for locally advanced EC patients [3,4,127,128]. The updated CROSS study has shown that the median overall survival was 48.6 months in patients receiving nCRT plus surgery cohort and 24.0 months in the surgery cohort (*p* = 0.030) [128]. The recent NEOCRTEC5010 trial also showed that EC patients receiving nCRT followed by surgery have a significantly increased median overall survival than those receiving surgery alone (100.1 vs. 66.5 months, *p* = 0.025) [4]. But patients have different responses to nCRT treatment, which significantly affects the survival outcomes. According to a recent review by Eyck et al. [129], the ability of commonly used imaging modalities to detect pCR after nCRT of EC patients was insufficient, indicated by the pooled sensitivities and specificities. An accurate estimation of residual disease could improve patient management.

For this purpose, there has been an increasing number of studies in the literature that utilized new methods for more accurate prediction. In our review article, we assessed the potential of ML approaches for a more accurate evaluation of pCR. This showed that ML models evaluating treatment response achieved reliable predictions ranging from acceptable (AUC = 0.70 to 0.80, *n* = 3) to excellent (AUC = 0.80 to 0.90, *n* = 7), and outstanding (AUC > 0.90, *n* = 1) in the validation group. The reviewed studies adopted a variety of ML approaches. Advanced imaging features were extracted using radiomics calculation algorithm or transfer learning technique from various pre-trained CNNs. According to Hu’s study characterizing relatively large sample sizes with extrapolation of developed models by external validation, the model using deep learning features extracted from Resnet had a better performance than the handcrafted radiomics model [83]. It is suggested that non-invasive ML-based imaging applications, such as radiomic techniques and deep learning networks are potentially useful for individualized patient’s tumor characterization.

Patients’ risk stratification mainly relies on the American Joint Committee on Cancer (AJCC) tumor, node, and metastasis (TNM) staging classification of epithelial cancers of the esophagus. Improvement of the staging method will aid in the current clinical practice. Involvement of lymph node invasion has been proved to be an important prognostic factor for both esophageal SCC and adenocarcinoma [130,131]. Recent refinement of the N descriptors (regional lymph node invasion) subcategory in the eighth edition has been shown to provide a more accurate and reliable risk stratification of EC patients [132]. Compared with the seventh edition, this new N subcategory excludes some regional lymph node stations common in the staging system of lung cancers [133]. Determination of clinical N (regional lymph node invasion) mainly relies on current imaging techniques. CT, one of the most commonly used non-invasive imaging techniques is often initially used for the evaluation of tumor growth and other structures. However, it is not optimal for lymph node determination [134,135]. Besides, the efficacy of MRI for the accurate estimation of N staging is still uncertain [136].

Improvement in imaging assessment of regional lymph nodes by using ML techniques is of clinical importance for prognosis and treatment decisions for EC patients. Radiomics, as an emerging tool, has shown potential values in predicting LN metastasis by extracting high-throughput quantitative features from medical images. Less attention has been paid to the prediction of LN metastasis in previous years. With the first study published in 2018, specific attention is paid to the evaluation of LN metastasis in EC patients. The reviewed studies [118,119,120] were mainly focused on the application of radiomics and Wu et al. [88] showed the prediction value of deep learning features for LN metastasis. According to our review, the new ML approach significantly improved the evaluation of N status using non-invasive CT and MRI modalities (AUC = 0.762–0.840 in the validation set), even outperforming the size criteria [88,118,119,120]. However, current published studies were limited to binary discrimination of LN status. The specific N staging information (N0/N1/N2/3) determined by evidence acquired before treatment affects the therapy scheme. Moreover, the drainage of the lymph nodes in EC is more complicated than in some cancers like lung cancers and breast cancers. The regional lymph node stations for EC patients extended from cervical to celiac regions, and the lymphatic drainages could be different at different anatomic sites. An optimal lymphadenectomy is necessary to maximize patients’ survival. The extent of lymphadenectomy is variable in current clinical practice [137,138]. Greater extent of lymphadenectomy was associated with patients’ long-term survival and its effect on postoperative complications [139,140]. The evaluation of individual LN requires laborious and time-consuming work from experienced radiologists. More accurate evaluation of more precise N staging or individual lymph node status could lead to a more precise assessment of the ideal range of lymph node dissection.

In our review, we have uncovered several shortcomings of existing clinical applications using machine learning. We will discuss these issues more broadly and then more specifically in the following paragraphs. The topic of explainability has been shown to be an important aspect of ML research [141]. Doctors may find it difficult to apply ML models routinely. CNNs, for example, could contain millions of trainable parameters without clearly understandable biological patterns for human readers. It is crucial that clinicians and patients have the ability to understand the reasonings of predictions of these models for better informed decision-making. The accountability of ML approaches is of vital importance if we introduce computerized ML systems into clinical practice. Currently, there is a paucity in the understanding of the relationship between radiomics and underlying tumor biology. The exact relationship of a combination of radiomics features used in the predictive modelling remains unclear. Further exploration has been attempted by, for example, doing radiogenomics studies, which were done in a few reviewed studies, providing proof of potential biological explanation. The reliability of the established prediction models could also be validated by statistical methods to reduce the concerns regarding applicability. To this end, some models were validated by statistical analysis using model fitting, calibration, and clinical usefulness. Simple data-driven correlation may not be robust and links between imaging, clinical, and genetic features should be built. Note that the precise relationship between imaging features and tumor heterogeneity is not simply straightforward. Further investigation of the advanced statistical relationship should be established. Visualization is suggested to be employed to improve the interpretability of ML models. More work should be dedicated to making ML models more interpretable or explainable for decision-making.

A few more specific limitations are noted from our review. Firstly, ML algorithms utilized in some studies were trained and validated on the same dataset, which is not an accurate estimation of ML models. Secondly, the sample size is one of the vital factors for clinical studies, which could significantly affect the repeatability, reproducibility, and statistical power. Adequate sample size is needed to train and test the ML methods to minimize overfitting and improve the estimation performance. The sample size criteria for some ML studies for EC patients were established with less than 100 patients, which limits the reliability of the proposed models. Clinically meaningful size is required for significant discrimination of patients in different risk groups. Thirdly, the reference standard for each outcome in question may be different, reflecting the heterogeneity of the study design impacting comparison. Most studies evaluating treatment response, for example, compared their model with histology findings by pathologists, which is the current gold standard. However, some studies [100,103,105,108] used pre-treatment and post-treatment CT images to assess the response based on the Response Evaluation Criteria in Solid Tumors (RECIST) [142], when resected specimens were not available. RECIST is commonly used for residual disease evaluation for solid tumors but has limited ability in EC for its obscure boundaries or scarred tissues after chemoradiation therapy [143]. Fourthly, standardization in imaging protocols (for example, CT with or without intravenous contrast), radiomics extraction methods, and attempts to harmonize imaging data prior to predictive modelling were scantily performed. These have been known to affect the extracted features and remain major obstacles to generalizability.

We have a few recommendations for future investigations. Firstly, prospective large multi-center studies should be performed to improve the ML techniques and generalizability with standardized imaging protocols and harmonization between different centers. In our review, only six studies [83,88,100,101,114,115] included multi-center datasets. Secondly, the techniques of machine learning analysis should be standardized. More strict adherence to the standard working pipeline as well as openly available source code is suggested to increase reproducibility and generalizability, particularly for radiomics features pipelines [144]. Thirdly, the application of ML methods should be expanded from restricted data-processing computers to portable machines accessible by cloud services or to intraoperative decisions in real-time. There are some challenges to overcome such as automated segmentation and real-time inference processing which currently limits its wider utility in the clinical environment. If this could be done, it would allow for prospective study design, and if randomized, it could add significant value to the assessment of the utility of these techniques in real-world clinical practices. Finally, the discrimination impact using these models needs to be clinically meaningful. Several studies based their performance assessment on AUC. Clinically meaningful size effects like decision curve analysis should be included more routinely.

## 5. Conclusions

Our review has summarized the predictive performance of non-invasive imaging ML applications in EC patients. Recent advances and future perspectives of the ML technique demonstrate its potential to provide novel quantitative imaging markers in medical imaging. A few recommendations are made to improve study design and future applicability.

## Figures and Tables

**Table 1:** Characteristics of studies for non-invasive imaging machine learning applications in esophageal cancers.

Studies	Year	Type	Treatment Regime	Approach	Modality	Sample Size (Training + Testing)	Ml Techniques	Classifiers for The Final Model	Specific Predicted Clinical Outcome	Type of Validation	Main Results (in Test Set)	Reference Standard
**Treatment response**												
Cao et al. [100]	2020	All SCC	CRT	Radiomics	PET	159 (93 + 66)	LASSO	LASSO	pCR	External validation	AUC = 0.835	CT
Hu et al. [101]	2020a	All SCC	nCRT followed by surgery	Radiomics	CT	231 (161 + 70)	Decision tree, recursive feature addition, LR, SVM, K-nearest neighbors, naive bayes, decision tree, RF, and extreme gradient boosting	SVM	pCR	External validation	AUC = 0.852 (95% CI, 0.753–0.951), accuracy = 84.3%, Se = 90.3%, Sp = 79.5%	Histology
Hu et al. [83]	2020b	All SCC	nCRT followed by surgery	Radiomics and deep learning	CT	231 (161 + 70)	Xception, VGG16, VGG19, ResNet50, InceptionV3, InceptionResNetV2, recursive feature addition, SVM	ResNet50-SVM	pCR	External validation	AUC = 0.805 (95% CI, 0.696–0.913), accuracy = 77.1%, Se = 83.9%, Sp = 71.8%	Histology
Yang et al. [102]	2019a	All SCC	nCRT followed by surgery	Radiomics	CT	55 (44 + 11)	LASSO	LR	pCR	Training + testing set (randomly separated)	AUC = 0.79 (95% CI, 0.48 to 1.00)	Histology
Hou et al. [103]	2018	All SCC	CRT	Radiomics	MRI	68 (43 + 25)	SVM and ANN	ANN	pCR	Training + testing set (randomly separated)	AUC = 0.843, accuracy = 84.3%, Sp = 100%	CT/MRI
Beukinga et al. [104]	2018	Adenocarcinoma 89.0%, SCC 11.0%	nCRT followed by surgery	Radiomics	PET	73	LASSO	LR	pCR	No validation	AUC = 0.81	Histology
Hou et al. [105]	2017	All SCC	CRT	Radiomics	CT	49 (37 + 12)	SVM and ANN	ANN	pCR	Training + testing set (randomly separated)	AUC = 0.800, accuracy = 91.7%	CT
Van Rossum et al. [106]	2016	All adenocarcinoma	nCRT followed by surgery	Radiomics	PET	217	LR	LR	pCR	Training + testing set (randomly separated, bootstrap method, repeated 1000 time)	C-index = 0.77 (95%, 0.70–0.83), Se = 0.78	Histology
Beukinga et al. [107]	2016	Adenocarcinoma 90.7%, SCC 9.3%	nCRT followed by surgery	Radiomics	PET-CT	97	LASSO	LR	pCR	No validation	AUC = 0.74	Histology
Desbordes et al. [108]	2016	Adenocarcinoma 12%, SCC 88%	nCRT followed by surgery or CRT	Radiomics	PET	65	Hierarchical forward selection method, RF, SVM	RF	pCR	Training + testing set (randomly separated, repeated 10 times)	AUC = 0.836 ± 0.105 (mean ± SD), Se = 82 ± 9%, Sp = 91 ± 12%	Follow-up based on clinical examination, endoscopy with biopsies and PET/CT
Ypsilantis et al. [109]	2015	Adenocarcinoma 81.1%, SCC 18.9%	nCRT followed by surgery	Radiomics and deep learning	PET	107 (96 + 11)	LR, gradient boosting, RF, SVM, 1S-CNN, 3S-CNN	3S-CNN	pCR	10-fold cross validation	Averaged Se = 80.7%, Sp = 81.6%	Histology
Zhang et al. [110]	2013	Adenocarcinoma 85%, SCC 15%	nCRT followed by surgery	Radiomics	PET	20	SVM and LR	SVM	pCR	10-fold cross validation	Averaged AUC = 1.00, Se = 100%, Sp = 100%	Histology
**Prognosis**												
Qiu et al. [111]	2020	All SCC	nCRT followed by surgery	Radiomics	CT	206 (146 + 60)	LASSO	Cox proportional hazards model	RFS	Training + testing (temporally separated)	Radiomics signature was significantly associated with RFS (log-rank test, *p* < 0.0001; HR, 3.606; 95% CI, 1.742–7.464). Radiomics nomogram C-index 0.724 (log-rank test, *p* < 0.001; 95% CI, 0.696–0.752)	Follow-up
Chen et al. [112]	2019	All SCC	nCRT followed by surgery	Radiomics	PET	44 (22 + 22)	LR	Cox proportional hazards model	OS and DFS	Training + testing set (randomly separated)	Significant risk stratification for DFS (log-rank test, *p* = 0.001) and OS (log-rank test, *p* < 0.001)	Follow-up
Yang et al. [113]	2019b	All SCC	Not specified to one kind of treatments	Deep learning	PET	model 1: 1107 (798 + 309), model 2: 548	3D-CNN based on ResNet	3D-CNN based on ResNet	OS	5-fold cross validation	The prediction result remained an independent prognostic factor (multivariable overall survival analysis, hazard ratio: 2.83, *p* < 0.001).	Follow-up
Xie et al. [114]	2019	All SCC	CRT	Radiomics	CT	133 (87 + 46)	The K-means method, LASSO	Cox proportional hazards model	OS	External validation	Prediction model AUC, 0.805 (95% CI: 0.638–0.973). Significant risk stratification (log-rank test, *p* < 0.001)	Follow-up
Larue et al. [115]	2018	Adenocarcinoma 81%, SCC 19%	nCRT followed by surgery	Radiomics	CT	239 (165 + 74)	Recursive feature elimination, RF	RF	OS	External validation	Prediction model AUC: 0.61 (95% CI: 0.47–0.75). Borderline significant risk stratification (log-rank test, *p* = 0.053)	Follow-up
Foley et al. [116]	2017	Adenocarcinoma 78.4%, SCC 21.6%	Not specified to one kind of treatments	Radiomics	PET	403 (302 + 101)	Automatic Decision Tree Learning Algorithm for Advanced Segmentation	Cox Regression Model	OS	Training + testing (temporally separated)	Significant risk stratification (log-rank test, *p* < 0.001)	Follow-up
Xiong et al. [117]	2018	SCC	CRT	Radiomics	PET	30	RF, SVM, LR and extreme learning machine	RF	PFS	Leave-one-out cross validation	Prediction model accuracy = 93.3%, Sp = 95.7%, Se = 85.7%. Significant risk stratification: (log-rank test, *p* < 0.001)	Follow up
**Lymph Node Metastasis**												
Wu et al. [88]	2020	All SCC	Surgery alone	Radiomics, computer vision, and deep learning	CT	411 (321 + 90)	Random Forest-Recursive Feature Elimination algorithm	LR	LN-positive versus LN-negative	External validation	AUC = 0.840	Histology
Qu et al. [118]	2018	Not stated	Surgery alone	Radiomics	MRI	181 (90 + 91)	Elastic net approach (a combination of the LASSO and the ridge regression approaches)	LR	LN-positive versus LN-negative	Training + testing (temporally separated)	AUC = 0.762 (95% CI: 0.713–0.812).	Histology
Tan et al. [119]	2018	All SCC	Surgery alone	Radiomics	CT	230(154 + 76)	LASSO	LR	LN-positive versus LN-negative	Training + testing set (randomly separated)	AUC = 0.773 (95% CI: 0.666–0.880)	Histology
Shen et al. [120]	2018	Not stated	Surgery alone	Radiomics	CT	197 (140 + 57)	Elastic net approach (a combination of the LASSO and the ridge regression approaches)	LR	LN-positive versus LN-negative	Training + testing (temporally separated)	AUC = 0.771 (95% CI: 0.632–0.910)	Histology
**Diagnosis**												
Li et al. [121]	2020	All SCC	Surgery alone (T3 cases) or no treatment (non-disease controls)	Radiomics	CT	57	Unspecified	LR	Malignant versus normal esophageal wall	No validation	AUC = 0.80	Histology
Ou et al. [122]	2019	All SCC	Not specified to one kind of treatments	Radiomics	CT	591 (413 + 178)	LASSO, LR, decision tree, random forest, SVM, and X-Gradient boost	LR	Resectability	Training + testing set (randomly separated)	AUC = 0.87 ± 0.02; accuracy = 0.86, and F-1score = 0.86	NCCN guidelines
**Gene expression**												
Hoshino et al. [123]	2020	All SCC	Not specified to one kind of treatments	Radiomics	CT	92	LR	LR	Expression of microRNA-1246	No validation	AUC = 0.754, Se = 71.29%, Sp = 73.91%	Follow-up

SCC: squamous cell carcinoma; CT: computed tomography; PET: positron emission tomography; MRI: magnetic resonance imaging; LASSO: least absolute shrinkage and selection operator; LR: logistic regression; SVM: support vector machine; RF: random forest; pCR pathologic complete response; AUC: area under the curve; CI: confidence interval; SD: standard deviation; ANN: artificial neural network; CNN: convolutional neural network; RFS: recurrence-free survival; PFS: progression-free survival; CRT: chemoradiation therapy; nCRT: neoadjuvant chemoradiation therapy; DFS: disease-free survival; OS: overall survival; Se: sensitivity; Sp: specificity; LN: lymph node; NCCN: National Comprehensive Cancer Network.

## Data Availability

Primary data cited in this review are openly available in PubMed, Embase and Cochrane database.

## Supplementary Materials

The following are available online at https://www.mdpi.com/article/10.3390/cancers13102469/s1, Table S1: Systematic search.
