# Supplementary material for: Machine Learning and Radiomics Applications in Esophageal Cancers Using Non-Invasive Imaging Methods—A Critical Review of Literature
Source: Cancers (Basel). 2021 May 19;13(10):2469. doi: 10.3390/cancers13102469 (PMC8158761; doi:10.3390/cancers13102469)
Supplement: Supplementary file 1 [file cancers-13-02469-s001.zip › Supplementary R v1.7.pdf]

Supplementary Table 1 – Systematic search

a) Medline

1. Esophageal Neoplasms/

2.(((((((((((((((Esophageal Neoplasm[Title/Abstract]) OR (Neoplasm, Esophageal[Title/Abstract])) OR (Esophagus Neoplasm[Title/Abstract])) OR (Esophagus Neoplasms[Title/Abstract])) OR (Neoplasm, Esophagus[Title/Abstract])) OR (Neoplasms, Esophagus[Title/Abstract])) OR (Neoplasms, Esophageal[Title/Abstract])) OR (Cancer of Esophagus[Title/Abstract])) OR (Cancer of the Esophagus[Title/Abstract])) OR (Esophagus Cancer[Title/Abstract])) OR (Cancer, Esophagus[Title/Abstract])) OR (Cancers, Esophagus[Title/Abstract])) OR (Esophagus Cancers[Title/Abstract])) OR (Esophageal Cancer[Title/Abstract])) OR (Cancer, Esophageal[Title/Abstract])) OR (Cancers, Esophageal[Title/Abstract])) OR (Esophageal Cancers[Title/Abstract])

3. 1 or 2

4. Machine learning/

5. Artificial Intelligence/

6. ((((((((((((((((((((((((Artificial Intelligence[Title/Abstract]) OR (Intelligence, Artificial[Title/Abstract])) OR (Computational Intelligence[Title/Abstract])) OR (Intelligence, Computational[Title/Abstract])) OR (Machine Intelligence[Title/Abstract])) OR (Intelligence, Machine[Title/Abstract])) OR (AI[Title/Abstract])) OR (Learning, Machine[Title/Abstract])) OR (Transfer Learning[Title/Abstract])) OR (Learning, Transfer[Title/Abstract])) OR (Radiomics[Title/Abstract])) OR (Radiomic[Title/Abstract])) OR (Radiogenomics[Title/Abstract])) OR (Radiogenomic[Title/Abstract])) OR (Support Vector Machine[Title/Abstract])) OR (SVM[Title/Abstract])) OR (neural networks[Title/Abstract])) OR (convolutional[Title/Abstract])) OR (CNN[Title/Abstract])) OR (deep learning[Title/Abstract])) OR (bayesian[Title/Abstract])) OR (network[Title/Abstract])) OR (classification[Title/Abstract])) OR (random forest[Title/Abstract])) OR (KNN[Title/Abstract])) OR (K nearest neighbors[Title/Abstract])) OR (decision tree[Title/Abstract])

7. 4 or 5 or 6

8.Multimodal Imaging/

9.Tomography, X-Ray Computed/

10.Positron-Emission Tomography/

11.Positron Emission Tomography Computed Tomography/

12.Magnetic Resonance Imaging/

13.Ultrasonography/

14.Diagnostic Imaging/

15.Radiology/

16.((((((((((((((((PET[Title/Abstract]) OR (pet-ct[Title/Abstract])) OR (FDG[Title/Abstract])) OR (positron[Title/Abstract])) OR (emission[Title/Abstract])) OR (tomography[Title/Abstract])) OR (EUS[Title/Abstract])) OR (endoscopic[Title/Abstract])) OR (ultrasound[Title/Abstract])) OR (ultraso*[Title/Abstract])) OR (MR*[Title/Abstract])) OR (magneti*[Title/Abstract])) OR (magnetic resonance imaging[Title/Abstract])) OR (CT[Title/Abstract])) OR (computed tomography[Title/Abstract])) OR (radiology[Title/Abstract])) OR (imaging[Title/Abstract])

17. 8 or 9 or 10 or 11 or 12 or 13 or 14 or 15 or 16

18. 3 and 7 and 17

2) Embase

1. exp esophagus tumor/
2. ((esophageal or esophagus or oesophageal or oesophagus) adj3 (cancer or cancers or tumor or tumour or tumors or tumours or neoplasm or neoplasms or malignancy or malignancies or adenocarcinoma or adenocarcinomas or carcinoma or carcinomas)).ti,ab,kw.
3. 1 or 2
4. exp Machine learning/
5. exp Artificial Intelligence/
6. (Artificial Intelligence or computational Intelligence or Machine Intelligence or Transfer Learning or Radiomics or Radiomic or Radiogenomics or Radiogenomic or Support Vector Machine or SVM or neural networks or convolutional or CNN or deep learning or Bayesian or network or classification or random forest or KNN or nearest neighbors or decision tree).ti,ab,kw.
7. 4 or 5 or 6
8. exp imaging/
9. exp computer assisted tomography/
10. exp positron emission tomography/
11. exp positron emission tomography-computed tomography/
12.exp Magnetic Resonance Imaging/
13. exp ultrasound/
14.exp radiology/
15. (PET or pet-ct or FDG or positron or emission or tomography or EUS or ultraso* or ultrasonography or MR* or magneti* or magnetic resonance imaging or CT or computed tomography or (computed adj tomography) or imaging or radiology).ti,ab,kw.
16. 8 or 9 or 10 or 11 or 12 or 13 or 14 or 15
17. 3 and 7 and 16

3) Cochrane

1. esophageal or esophagus or oesophageal or oesophagus
2. Artificial Intelligence or Machine learning or computational Intelligence or Machine Intelligence or Transfer Learning or Radiomics or Radiomic or Radiogenomics or Radiogenomic or Support Vector Machine or SVM or neural networks or convolutional or CNN or deep learning or Bayesian or network or classification or random forest or KNN or nearest neighbors or decision tree
3. PET or pet-ct or FDG or positron or emission or tomography or EUS or endoscopic ultrasound or ultraso* or ultrasonography or MR* or magneti* or magnetic resonance imaging or CT or computed tomography or tomography or imaging or radiology
4. #1 AND #2 AND #3
